# Commentary: An Adaptation-Induced Repulsion Illusion in Tactile Spatial Perception

**DOI:** 10.3389/fnhum.2018.00160

**Published:** 2018-04-24

**Authors:** Jack Brooks

**Affiliations:** Neuroscience Research Australia, University of New South Wales, Sydney, NSW, Australia

**Keywords:** somatosensory, tactile distance, tactile aftereffects, adaptation, object perception

Locating a stimulus on the skin is a well-described perceptual task, but little is known about how this ability remains stable under ecological conditions. Specifically, the effect of recent stimulation has been the subject of very few studies in the tactile domain (Craig, 1993; Braun et al., 2000), but is well described in the visual system. Exposure to lines at one orientation causes perceptual repulsion of the orientation of a subsequently viewed line (Gibson and Radner, 1937), an effect also observed in touch (Silver, 1969). In the tactile domain, contention surrounds a different kind of aftereffect, spatial repulsion, whereby adaptation can elicit a position shift of the perceived location of a stimulus away from the adapted area. Maintaining stable spatial perception in touch in these situations is important for tasks requiring fine motor control (Medina and Coslett, 2016; Goodman and Bensmaia, 2018), like the case of an artist switching between tools of different grip sizes, or a factory worker sorting and grading produce.

Day and Singer (1964) found a spatial repulsion aftereffect following sustained pressure on the forearm. Two bars placed either side of the adapted region were perceived as further apart than a comparison stimulus on the other arm. If the region outside of the bars was instead adapted, the perceived distance decreased. However, Gilbert (1967) found that this spatial distortion arose from a methodological bias from using an asymmetrical range of comparison stimuli. Given these results it remained open to investigation whether spatial repulsion is observed in the tactile domain and if so, by what neural mechanism.

In the study of Li et al. (2017) these questions were addressed over three experiments using a force-controlled two-point stimulus. The first experiment used a two-alternative forced choice task in which participants were given the two-point stimulus on the reference arm (always given 30 mm) and subsequently on the comparison arm (counterbalanced order). Critically, a symmetrical range of comparison stimuli was used (6, 12, 18, 24, 30, 36, 42, 48, or 54 mm). Participants were relatively stable at this task across time. In the second experiment adaptation was measured using a two-alternative forced choice touch detection task. Exposure to stimulation consisted of an initial 40 s of vibration with subsequent 3 s bursts of top-up stimulation separated by short breaks for threshold testing. Adaptation from the stimulation resulted in increased touch detection thresholds.The severity of the adaptation decreased with distance from the center of the stimulation. In the third experiment, vibro-tactile stimulation was applied to the area within the two points on the reference arm in the critical condition. Exposure was the same as in the second experiment, with the two-point comparison task performed in the breaks between top-up stimulation. The perceived distance of the two-point stimulus spanning the adapted area increased by approximately 10%.

In summary, the two preliminary experiments showed that tactile spatial perception is stable with time and that vibro-tactile stimulation reduces sensitivity to a subsequently applied touch stimulus. The main experiment found that adaptation results in perceived spatial expansion. Li et al. (2017) propose that the spatial distortion arises from changes in receptive field size, given two assumptions about the neural processing of tactile information. First, the brain remains largely unaware of peripheral adaptation (Seriès et al., 2009). Second, perceived location comes from the pooled response from multiple neurons (Ghazanfar et al., 2000). This hypothesis states that adaptation causes the signaled location of a touch to shift away from the adapted region, as there is a shift in the distribution of neural activity corresponding to the stimulus.

In a different test of such a model, my own study tested the effects of adaptation following exposure to tactile motion on the dorsal side of the forearm. The study used a 15 cm/s motion stimulus which moved along a 19 cm path but only touched the skin for 4.5 cm at the proximal and distal ends of motion. Exposure to this stimulation resulted in an inwards position shift (i.e., repulsion from the stimulus) of a stimulus at the edge of the gap (Brooks et al., 2015). The size of the shift was approximately six percent of the gap size, comparable to the effect observed by Li et al. (2017). Together these observations are most consistent with changes to receptive field geometry, but whether these manifest sub-cortically or cortically is unknown. One test of this would be to apply local anaesthesia as the adapting stimulus, or use different adapting and test stimuli (e.g., selectively adapt a class of mechanoreceptors).

Li et al. have shown results for only one stimulus size of adaptation, but could the observed spatial expansion be size dependent? Calzolari et al. (2017) show that repetitive exposure to a small or large two-point extent stimulus on the hand causes a subsequently presented stimulus to feel larger or smaller respectively. Local changes in receptive field geometry are unlikely to explain the effect, as the adapting stimulus was applied at slightly different locations to reduce the possibility of local adaptation. The authors raise the possibility of reduced responses of distance specific neurons with tuning curves that overlap with the adaptor.

Rather than considering aftereffects to arise as a result of fatiguing neurons, they might represent the optimization of perception to the recent statistics of the environment. In vision there is increasing evidence that perception is optimized to statistical regularities of the environment (Seriès and Seitz, 2013). In touch, evidence in macaques (Clark et al., 1988; Wang et al., 1995) and in humans (Braun et al., 2000) suggests that the somatosensory cortex reorganizes in response to prolonged periods of tactile stimulation. Whether any such processes operate for shorter windows of sensory input in other brain regions remains an open question. One way in which the new results of Li et al. (2017) can be functionalized is under the hypothesis that the body is used as a tool to perceive external objects (Linkenauger et al., 2010; Van der Hoort and Ehrsson, 2016). Under this hypothesis, the rapid malleability of higher order body representations (Taylor-Clarke et al., 2004) can afford enhanced object perception. For instance, creating the visual illusion that the arm is larger improves tactile discrimination (Kennett et al., 2001) and increases perceived distances on the arm (Taylor-Clarke et al., 2004). Thus, the perceived elongation of a two-point stimulus in the Li et al. (2017) study affords higher resolution perception of objects touching the adapted region.

Unlike many previous studies, Li et al. (2017) show preliminary evidence that aftereffects following only seconds of stimulation may persist for up to 20 min. Extending the exposure to tactile stimulation can result in even longer aftereffects. Mechanical stimulation for minutes can lead to improvements in tactile discrimination for hours (Godde et al., 2000), and weeks of vibro-tactile stimulation elicit shifts in perceived touch location weeks after the cessation of stimulation (Craig, 1993). A possible application of these methods is to correct distorted body representations. For instance, stroke patients with damage to somatosensory areas of the brain mislocalize touch toward the middle of the hand (Rapp et al., 2002; White et al., 2010). This distortion likely arises from a reduced cortical representation of the hand, leading to more uncertainty (Rapp et al., 2002; White et al., 2010). If sustained tactile stimulation does improve perception then it might be viable as a treatment for these patients.

In summary, it is clear that sustained pressure leads to repulsive spatial aftereffects. Li et al. (2017) have shown that vibro-tactile stimulation elicits a spatial dilation of a two-point stimulus spanning the stimulated area. This result seems consistent with a probabilistic model in which changes in the population response of neurons, to which the brain is unaware, result in a spatial distortion. These new findings raise intriguing possibilities for further work to determine if the spatial distortion arises from sub-cortical or cortical adaptation, optimizes object perception, and can be harnessed for clinical use.

## Author contributions

The author confirms being the sole contributor of this work and approved it for publication.

### Conflict of interest statement

The author declares that the research was conducted in the absence of any commercial or financial relationships that could be construed as a potential conflict of interest.
